# Single Dose Study Assessing the Pharmacokinetic and Metabolic Profile of Alverine Citrate in Healthy Volunteers

**DOI:** 10.3389/fphar.2020.620451

**Published:** 2021-01-20

**Authors:** Simona Rizea-Savu, Simona Nicoleta Duna, Roxana Colette Sandulovici

**Affiliations:** ^1^ 3S-Pharmacological Consultation & Research GmbH, Harpstedt, Germany; ^2^ Faculty of Pharmacy, Titu Maiorescu University, Bucharest, Romania; ^3^ 3S-Pharmacological Consultation & Res. SRL, Bucharest, Romania

**Keywords:** alverine citrate, 4-hydroxy alverine, 4-hydroxy alverine glucuronide, N-desethyl alverine, pharmacokinetics, *in-vivo* metabolism

## Abstract

Alverine citrate is a spasmolytic commonly prescribed in conditions such as irritable bowel syndrome, painful diverticular disease of the colon, and primary dysmenorrhea. While clinical efficacy data on alverine alone or in combination with simethicone is freely available, surprisingly little information regarding the pharmacokinetics and metabolism of alverine can be found in literature. The first HPLC-MS/MS analytical protocol for determination of alverine parent, 4-hydroxy alverine, N-desethyl alverine and 4-hydroxy alverine glucuronide in human plasma was developed and validated. The two validated methods were used for analyzing plasma samples collected during an open label, non-comparative, single dose, one-period, one-treatment, pharmacokinetic and metabolic profile study of Spasmonal^®^ Forte 120 mg hard capsule, conducted in 12 fasting healthy male and female volunteers of Caucasian descent. The study confirmed previous suspicions that parent alverine is subject to high pharmacokinetic variability and also revealed that the metabolic process most susceptible to outlying performance in Caucasians is hydroxylation to the active metabolite 4-hydroxy alverine. Another interesting observation made is that alverine parent accounts for only 3%, whereas total 4-hydroxy alverine (free and conjugated) accounts for 94% of alverine-related moieties in circulation (based on comparisons of total exposure).

## Introduction

Alverine citrate is a spasmolytic with specific action on the smooth muscle of the alimentary tract and uterus, which, at therapeutic doses, does not affect the heart, blood vessels or tracheal muscle (Boudghène et al., 2001; Annaházi et al., 2014). It belongs to a class of antispasmodic drugs which reduce the sensitivity of smooth muscle contractile proteins to calcium. By selectively binding with 5-HT1A receptors it acts as an antagonist that reduces the visceral pronociceptive effect of serotonin (5-HT) (Antoine et al., 2001). Due to these properties, alverine is used for the relief of smooth muscle spasm in conditions such as irritable bowel syndrome, painful diverticular disease of the colon and primary dysmenorrhea (Annaházi et al., 2014).

An independent meta-analysis and systematic review of clinical trials data relating to the effect of antispasmodic agents, alone or in combination, in the treatment of Irritable Bowel Syndrome showed superiority of the alverine/simethicone combination over other antispasmodic agents such as pinaverium bromide, mebeverine, trimebutine, hyoscine, fenoverine and dicyclomine, in terms of both patient global assessment on symptoms relief and pain relief (Martínez-Vázquez et al., 2012).

Clinical efficacy data on alverine alone or in combination with simethicone is freely available (Wittmann et al., 2010; Martínez-Vázquez et al., 2012; Ducrotte et al., 2014). The sparse information regarding the metabolism of alverine available in scientific literature is solely centered on the pharmacokinetics of the parent compound and the active metabolite 4-hydroxy alverine (Gomes et al., 2009; Ghosh et al., 2010; Seelam et al., 2015; Rathod et al., 2017) and is only derived from studies conducted on Indian population, where intra-subject variability of the main pharmacokinetic parameters of the parent compound seems quite low based on the required sample size for successful demonstration of bioequivalence between distinct oral dosage forms.

In terms of data reviewed by the European Medicines Agency and found to be representative for the general population, the summary of product characteristics of Spasmonal (SmPC Spasmonal Forte 120 mg, 2018) briefly describes that after oral administration of alverine, it is rapidly converted to its primary active metabolite (T_max_ between 1 and 1 ½ hours after dosing) and the latter is then converted to two secondary (unnamed) metabolites. Renal clearance is reported as high for all metabolites, indicating that they are eliminated by active renal secretion.

The present study aimed to contribute more concrete information with respect to the pharmacokinetic and metabolic profile of alverine citrate in Caucasian population, by simultaneous quantification of the parent compond, the active metabolite and the two secondary metabolites, following the administration of a single oral dose of the marketed reference product Spasmonal^®^ Forte 120 mg hard capsule.

## Materials and Methods

### Standards and Reagents

The reference standards of alverine citrate (99.2% purity), 4-hydroxy alverine hydrochloride (98.3% purity), 4-hydroxy alverine glucuronide (98.6% purity), N-desethyl alverine hydrochloride (99.9% purity) and the internal standards (IS) D5-alverine citrate (99.2% purity) and D5-4-hydroxy alverine hydrochloride (98.2% purity) were obtained from TLC Pharmaceutical Standards (Ontario, Canada). The internal standard buprenorphine-3-β-D-glucuronide (98.5% purity) was sourced from Cerilliant Analytical Reference Standards (Texas, USA). Methanol, acetonitrile and ammonium formate were of high-performance liquid chromatography (HPLC) grade, purchased from Merck (Darmstadt, Germany). Water was purified using Milli-Q water purification system from Millipore.

### Equipment

A Shimadzu HPLC system, consisting of a CTC autosampler, LC-20AD binary pump, DGU-20A5 degassing unit, and CTO-20AC thermostatted column oven (Shimadzu, Kyoto, Japan), were used for the validation tests as well as real samples analysis. The mass spectrometer utilized for this work was a Sciex API 5500 triple-quadrupole mass spectrometer equipped with atmospheric pressure electrospray ionization interface (Turbo V) (AB Sciex, Foster City CA). Study data were collected using Analyst® (Version 1.7 Applied Biosystems). MPX Driver (using MPX SW version 2.0) software was used to control the LC parameters.

### Liquid Chromatography and Mass Spectrometric Conditions

Two separate analytical methods were developed, validated, and used for real samples analysis: the first one (from here on referred to as Method A) allowed for the simultaneous quantification of alverine parent, 4-hydroxy alverine and N-desethyl alverine; the second one (from here on referred to as Method B) was used for quantification of 4-hydroxy alverine glucuronide. All chromatographic separations were carried out using Discovery C18 (12.5 cm × 2.1 mm; 5 µm) silica packing reversed phase analytical columns. For both methods the mobile phase consisted of 10 mM ammonium formate in water (pH 4.5) and acetonitrile. Samples of 40 µl were loaded onto the column, separated and eluted in gradient conditions. The total run time was 4.5 min for Method A and respectively 3.5 min for Method B. In both cases the autosampler temperature was held at 10°C and the mass spectrometer was run in positive ion ESI mode using multiple-reaction monitoring (MRM) to monitor the mass transitions. Research grade nitrogen was used as curtain gas and collision gas (CAD). The resolutions for both Q1 and Q3 were set at unit. A summary of the ion transitions, declustering potentials, collision energies, and collision cell exit potentials are presented for both methods in Table 1.

**TABLE 1:** Optimal positive ion ESI mass spectrometric conditions for multiple reaction monitoring.

Method	Analyte	Ion transition	Dwell time (ms)	Declustering potential (V)	Collision energy (V)	Collision cell exit potential (V)
A	Alverine	282.194 >> 91.200	75	200	37	24
	D5-alverine (IS)	287.230 >> 91.200	75	200	53	24
	4-Hydroxy alverine	298.169 >> 106.900	75	200	37	16
	D5-4-hydroxy alverine (IS)	303.685 >> 106.600	75	200	39	12
	N-desethyl alverine	253.989 >> 90.600	75	200	35	10
B	4-Hydroxy alverine glucuronide	474.256 >> 298.000	150	100	39	32
	Buprenorphine-3-β-D-glucuronide (IS)	644.259 >> 467.900	150	100	53	40

### Calibration Curves and Quality Control Samples

Stock solutions of each analyte (alverine, 4-hydroxy alverine, N-desethyl alverine and 4-hydroxy alverine glucuronide) were prepared in DMSO at a concentration of 1.000 mg/ml. The stock solutions of internal standards for Method A (d5-alverine and d5-4-hydroxy alverine) were prepared at 1.000 mg/ml and for Method B (buprenorphine-3-β-D-glucuronide**)** at 500.000 ng/mL, in methanol. These solutions were stored at −20°C. A series of working solutions for preparation of the eight points calibration curves and the plasma QC samples were obtained by mixing and diluting the stock solutions with pooled human plasma deriving from blank blood samples collected on Li-heparin from healthy volunteers. The concentrations of the spiked QC samples and the range of the calibration curves by analyte are presented in Table 2.

**TABLE 2:** Concentrations of the QC samples and range of the calibration curves by analyte.

Method	Analyte	Calibration range		QC samples
		LLOQ	ULOQ	
A	Alverine	20 pg/ml	15,000 pg/ml	60–1,000 - 6,000–12,000 pg/ml
	4-Hydroxy alverine	20 pg/ml	15,000 pg/ml	60–1,000 - 6,000–12,000 pg/ml
	N-desethyl alverine	20 pg/ml	15,000 pg/ml	60–1,000 - 6,000–12,000 pg/ml
B	4-Hydroxy alverine glucuronide	1 ng/ml	750 ng/ml	3–50 - 300–600 ng/ml

### Pharmacokinetic Study in Healthy Volunteers

The validated methods were used for analysis of real samples from an open label, non-comparative, single dose pharmacokinetic and metabolic profile study of Spasmonal^®^ Forte 120 mg hard capsule (containing 120 mg alverine citrate), manufactured by MEDA Pharmaceuticals Ltd, UK under license of Mylan Products Limited, UK, sourced from the UK market.

The study included 12 fasting healthy male and female volunteers, Caucasian (Eastern Europeans of Moldavian nationality), adults (between 18 and 55 years of age) with a body mass index within 18.5–30.0 kg/m^2^. All subjects gave their written informed consent before they underwent any study-related procedures and were free to withdraw from the trial at any time. The study medication administration consisted of one single 120 mg hard capsule of alverine citrate (trade name: Spasmonal^®^ Forte) taken orally with 200 ml of still bottled water after at least 10 h of overnight fasting. For the analytical determination of alverine, hydroxy alverine (free and conjugated) and N-desethyl alverine plasma levels, venous blood samples of 5 ml were drawn in tubes containing Li-heparin as anticoagulant before study drug administration and at 0.25, 0.5, 0.75, 1.0, 1.5, 2.0, 2.5, 3.0, 4.0, 5.0, 6.0, 8.0, 10.0, 12.0, 14.0, 16.0, 24.0, 30.0, 36.0, 48.0 and 72.0 h post dose. The pharmacokinetic parameters calculated for each analyte were AUC_0-t_, C_max_, T_max_, AUC_0-∞_, t_1/2_ and MRT. The study was conducted in the Republic of Moldavia following unconditional approval from the National Ethics Committee and the National Medicine Agency. Clinical investigations were conducted according to the Declaration of Helsinki principles and Good Clinical Practice.

### Handling of Study Samples

After collection, the blood samples were centrifuged under refrigeration (10 min at 1,500 (±5) g and a nominal temperature of 4°C). Plasma was separated, divided into duplicate aliquots and, within 60 min from collection, frozen for storage at −20°C nominal until shipped to the analytical laboratory. Plasma samples (first aliquot) were sent from the clinical site to the analytical facility in a thermo-insulated box containing an adequate amount of dry ice. During transport, an electronic logger was used for monitoring plasma samples temperature. Once received at the analytical laboratory, the samples were stored at −20°C or colder until submitted to analysis. Before analysis, plasma samples were thawed, mixed for 3 min and centrifuged for 3 min at 4,000 rpm and 20°C nominal. Aliquots of samples isolated for Analytical Method A were: spiked with internal standards (d5-alverine and d5-4-hydroxy alverine), diluted, vortexed and centrifuged; supernatants were evaporated to dryness under air stream, reconstructed with a water/acetonitrile solution (1/1, v/v), mixed and centrifuged; finally, the samples have been transferred in the autosampler to be injected. Aliquots of samples isolated for Analytical Method B were: spiked with internal standard (buprenorphine-3-β-D-glucuronide), diluted, vortexed and centrifuged; supernatants were diluted with water, mixed and centrifuged; finally, the samples have been transferred in the autosampler to be injected. Representative chromatograms for each analyte vs. the reference internal standard are presented as Supplementary Material.

The analytical work was performed according to GLP principles and FDA requirements (FDA, 2018 Bioanalytical Method Validation Guidance, 2018). The analytical methods were fully validated before starting the analysis of study plasma samples. The methods were verified for linearity, quantification limits, assay specificity, between-run and within-run precision and accuracy, analyte recovery, and stability in stock solution and biological matrix under processing conditions during the entire period of storage.

### Pharmacokinetic and Statistical Analysis

Non-compartmental PK analysis was performed using SAS^®^ statistical software, version 9.4 (SAS Institute Inc., Cary, North Carolina, USA). Maximum plasma concentration (C_max_) and time to reach maximum plasma concentration (T_max_) were obtained directly from the plasma values. The linear trapezoidal rule was used to calculate the area under the concentration-time curve from time zero to the last quantifiable concentration (AUC_0–t_). The apparent elimination rate constant (K_el_) was estimated by regression of the terminal ln-linear portion of the plasma concentration–time profile; apparent terminal half-life (t_*½*_) was calculated as the quotient of ln (2) and K_el_. Area under the curve to infinity (AUC_0–∞_) was estimated as the sum of AUC_0–t_ and the extrapolated area given by the quotient of the last quantifiable plasma concentration and K_el_. ANOVA was performed on ln-transformed C_max_, AUC_0-t_ and AUC_0–∞_ using the General Linear Models (GLM) procedure fitted in SAS^®^ software using the method of least squares. Descriptive statistics were performed for all pharmacokinetic parameters.

## Results

### Method Validation Results

#### Selectivity

Analyses were performed on 8 blank plasma samples from different healthy volunteers (including a lipemic and a hemolytic sample) without any addition and then with addition of internal standards or alverine or 4-hydroxy alverine or 4-hydroxy alverine glucuronide or N-desethyl alverine or possible co-medication; no peak, interfering with those of the analytes or the internal standards appeared in the blank samples. Same test was applied to all types of LLOQ samples. None of the blank plasma sources showed any obvious interference.

#### Calibration Curve: Fitting, Precision and Accuracy

The precision and the accuracy, at all concentrations, were satisfactory; the curve fittings were also optimal in the whole range with correlation coefficients (*r*) = 0.99790 for alverine (*r*) = 0.99740 for 4-hydroxy alverine (*r*) = 0.99740 for 4-hydroxy alverine glucuronide and (*r*) = 0.99750 for N-desethyl alverine.

#### Extraction Recovery

The extraction recoveries of QC samples, calculated on the peak areas of alverine (mean recovery across the three QC levels tested: 75%), 4-hydroxy alverine (mean recovery across the three QC levels tested: 84%), 4-hydroxy alverine glucuronide (mean recovery across the three QC levels tested: 87%) and N-desethyl alverine (mean recovery across the three QC levels tested: 76%) put in evidence that the extraction was effective at all tested concentrations with all compounds, being above 70.0% for all individual tests; therefore, adequate for an analytical method. The extraction recoveries of the internal standards were also effective, being above 70.0%, and adequate for a reliable analytical method.

#### Matrix Effect

The matrix effect was also evaluated. Matrix Factor (MF) extracted individual blank plasma samples (8 blank plasma samples from different healthy volunteers including a lipemic and a hemolytic sample for each concentration level) spiked with standard of extraction solutions in mobile phase at the concentrations of QC1 and QC4 (after extraction) were analyzed; the peak areas were compared to the same standard of extraction solution peak areas in mobile phase. The matrix factors obtained for alverine/d5-alverine, 4-hydroxy alverine/d5-4-hydroxy alverine, 4-hydroxy alverine glucuronide/buprenorphine glucuronide, and N-desethyl alverine/d5-alverine were slightly lower than one and suggest that no significant ionization suppression occurs in presence of matrix ions. The precision of the IS normalized matrix factors was always less than 15% and therefore adequate for reliable bio-analytical assay.

#### Carry-Over Effect

The carry-over effect was assessed by injecting blank samples after high concentrated samples (CAL 8) in six consecutive series. The analytes blank chromatographic response was supposed to be 5 times smaller than the one given by calibrator 1 samples. The internal standards blank chromatographic response was supposed to be 20 times smaller than the one given by the previous CAL8 sample. The results showed that no signal was detectable in blank samples injected after high concentrated samples (calibrator 8) and therefore can be concluded that no carry-over effect was present.

#### Spiked Plasma Samples Stability

The results obtained in all storage conditions show that alverine, 4-hydroxy alverine, 4-hydroxy alverine glucuronide and N-desethyl alverine are stable in plasma for the following durations: up to 6 h at room temperature (benchtop stability), up to 1 week at −5°C (autosampler stability), up to 3.5 weeks below −20°C (storage stability covering the timeframe from collection of the first sample to assay of the last sample) and up to 1 week below −70°C (transport stability fully covering the transit time from the clinic to the analytical laboratory).

#### Stability of Spiked Plasma Samples Extract

The results obtained show that alverine, 4-hydroxy alverine and N-desethyl alverine are stable in plasma extracts and dry extracts up to 96 h when kept at 10°C nominal and that 4-hydroxy alverine glucuronide is stable in plasma samples extract up to 48 h when kept at 10°C nominal (short-term refrigerated storage stability).

#### Dilution Test

Since there is always a chance that real samples have analytes levels exceeding the maximum concentrations of the calibration curves, a past-dilution method 1 + 19 with blank plasma was validated. The mean dilution accuracy was within the range of variation accepted for QC samples with all compounds.

#### Stock Solutions Stability

The stock solutions of the analytes and internal standards are stable up to 12 days when stored below −20°C and up to 6 h if kept at room temperature.

#### System Suitability Test Solution Stability

The suitability test solutions of the analytes and internal standards were stable up to 12 days when stored below −20°C.

#### Blood Sampling Tubes Validation

The risk of unreliable quantitation results was excluded for all analytes after testing the Monovette tubes (Sarstedt, Germany) pre-filled with lithium heparin as anticoagulant.

#### Plasma Hemolyzation Impact on Accuracy of Analytes Determination

The accuracies have been calculated from the samples prepared at QC3 concentration level at three hemolyzation levels (low, middle and high) and at QC1 concentration level (middle hemolyzation level). It was concluded that the measurement accuracy was not affected in hemolytic samples.

#### Interconversion (Conversion And/Or Back-Conversion) Tests

The interconversion of alverine, 4-hydroxy alverine, 4-hydroxy alverine glucuronide or N-desethyl alverine was evaluated both in plasma samples before analyses and during the analytical phase. The mean results at all tested concentrations were in range (±15% (85–115%) vs. nominal). The measurements were adequate in all tested conditions thus excluding the relevance of analytes interconversion.

From the results previously reported it can be concluded that the developed analytical methods had adequate sensitivity, precision, accuracy, and specificity to quantitatively determine alverine, 4-hydroxy alverine, 4-hydroxy alverine glucuronide or N-desethyl alverine together or separately at the concentrations expected in alverine clinical studies samples.

Concentrations in validation samples were estimated on the regression curves obtained from the data of the calibration samples run in the same sequence.

Calculations were carried out on alverine (chromatographic trace m/z 282.194/91.200) peak areas normalized to the internal standard (d5-alverine) peak areas (chromatographic trace m/z 287.230/91.200) or 4- hydroxy alverine (chromatographic trace m/z 298.169/106.900) peak areas normalized to the internal standard (d5-4-hydroxy alverine) peak areas (chromatographic trace m/z 303.685/106.600) or 4- hydroxy alverine glucuronide (chromatographic trace m/z 474.256/298.000) peak areas normalized to the internal standard (buprenorphine-3-β-D-glucuronide) peak areas (chromatographic trace m/z 644.259/467.900) or N-desethyl alverine (chromatographic trace m/z 253.989/90.600) peak areas normalized to the internal standard (d5-alverine) peak areas (chromatographic trace m/z 287.230/91.200). The calculations of concentrations were performed using weighted (1/x2) linear regression models for alverine, 4-hydroxy alverine, and N-desethyl alverine and respectively (1/x) quadratic regression models for 4-hydroxy alverine glucuronide.

There were no interferences of endogenous compounds at the retention times of alverine, 4-hydroxy alverine, 4-hydroxy alverine glucuronide or N-desethyl alverine for double blank plasma, blank plasma, samples spiked at LLOQ concentration and subject samples at C_max_ and IS after oral administration.

### Pharmacokinetic Results

The need of performing a metabolic characterization of alverine citrate was identified after a close analysis of observations made in a previous pivotal fully-replicate design study conducted on a sample size of 42 healthy volunteers. At that time, analysis of parent alverine alone revealed that a number of subjects exhibited consistent (same pattern observed across study periods) subject-specific outlying pharmacokinetic trends which led to a huge inter-individual variability (coefficient of variation within treatment group of approximately 300% for both C_max_ and AUC_0-t_). Also, a very high within-subject variability of the primary PK parameters was noted within the replicate reference group (Reference intra-subject CV calculated by EMA Method A (EMA, 2011) for alverine C_max_ data for the purpose of applying scaled bioequivalence limits was 84%). In the absence of own metabolite data or revealing literature information to support the high variability observed, a more detailed pharmacokinetic profiling of alverine and its metabolites was pursued in the new exploratory study presented in this article.

A total of 12 healthy male and female volunteers were enrolled and completed the exploratory study. All subjects were Caucasian with the mean age of 33.42 years (range 18–55 years) and mean BMI of 25.13 kg/m^2^ (range 20.8–29.6 kg/m^2^).

**FIGURE 1:**
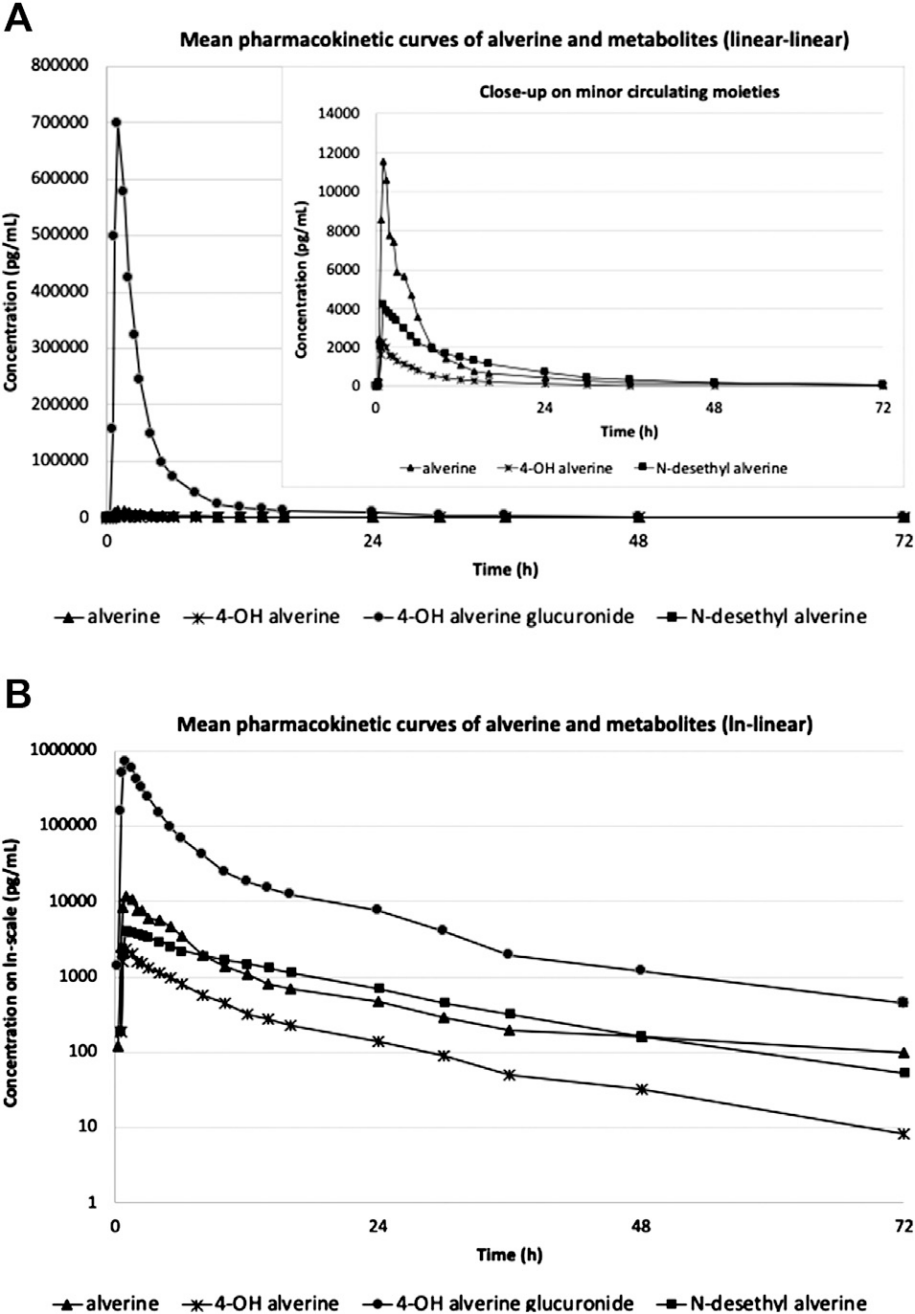
Mean pharmacokinetic curves of alverine parent and metabolites (*N* = 12) in linear-linear display **(A)** and ln-linear display **(B)**.

The mean alverine, 4-hydroxy alverine, N-desethyl alverine and 4-hydroxy alverine glucuronide concentration-time curves are shown in Figure 1 (linear-linear display in panel A and ln-linear display in panel B) while mean pharmacokinetic parameters are summarized in Table 3.

**TABLE 3:** Mean Pharmacokinetic Parameters after a single dose of Spasmonal^®^ Forte 120 mg hard capsule, administered to fasting healthy volunteers (N = 12). Presented in bold are the mean values.

PK parameter	Alverine	4-OH alverine	4-OH alverine glucuronide	N-desethyl alverine
**C**_**max**_ **(pg/ml)** Mean (CV %)	**12,967** (219%)	**2,871** (123%)	**752,123** (35%)	**4,329** (183%)
**AUC**_**0-t**_ (pg/ml*h) Mean (CV %)	**64,743** (209%)	**15,336** (111%)	**1,996,700** (45%)	**51,746** (163%)
**AUC**_**0-∞**_ (pg/ml*h) Mean (CV %)	**70,557** (221%)	**16,130** (108%)	**2,021,278** (44%)	**90,612** (133%)
**T**_**max**_ **(h)** Median (range)	**0.75** (0.50–1.50)	**1.00** (0.50–2.50)	**1.00** (0.75–1.50)	**1.00** (0.50–2.50)
**K**_**el**_ **(1/h)** Mean (CV %)	**0.08** (75%)	**0.04** (25%)	**0.10** (85%)	**0.15** (98%)
**t**_**½**_ **(h)** Mean (CV %)	**16.52** (103%)	**15.37** (38%)	**8.42** (62%)	**8.37** (70%)

**FIGURE 2:**
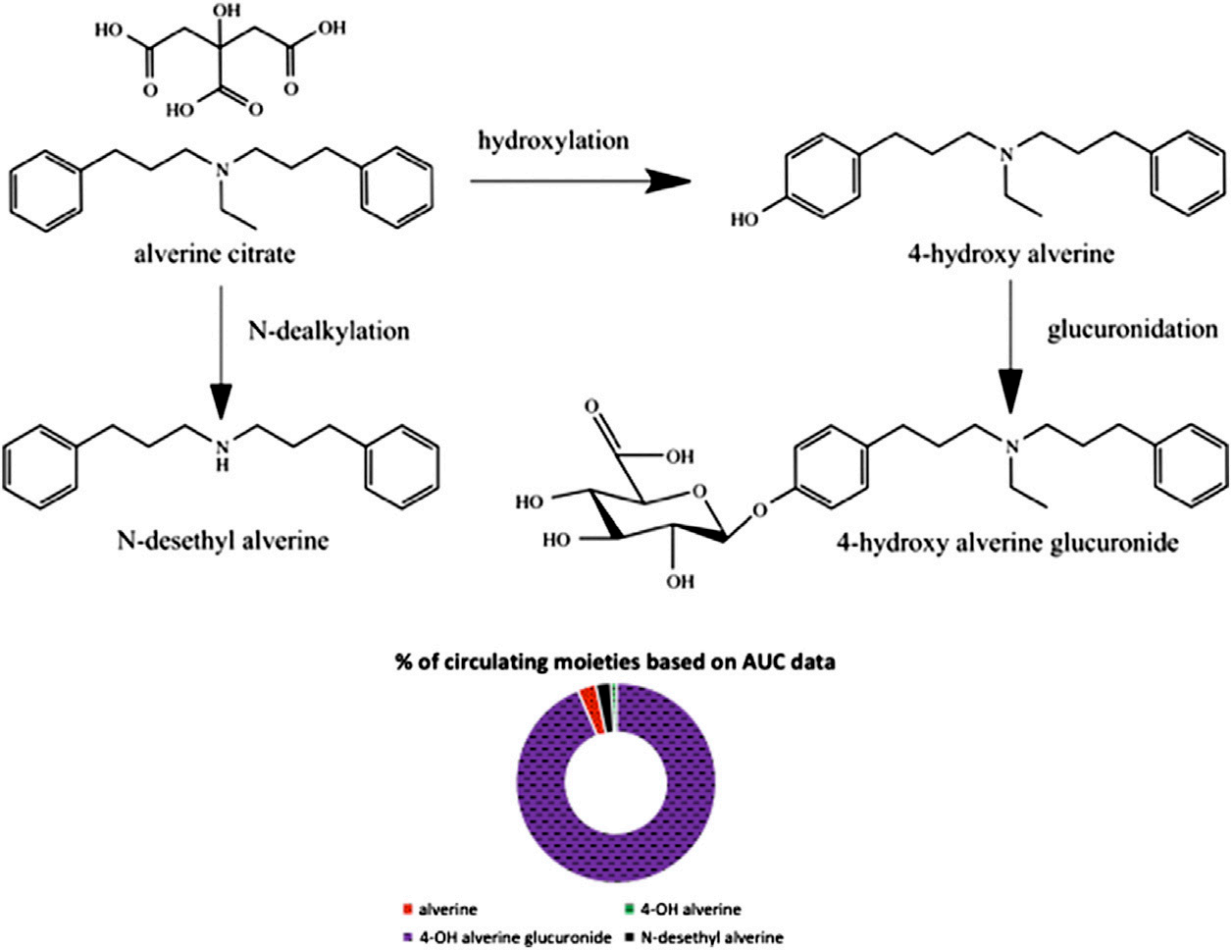
Schematic representation of metabolic pathways and individual contribution of each moiety to the overall alverine-related exposure.

A schematic representation of the metabolic pathways which lead to formation of the quantified moieties and the individual contributions of the respective moieties to the overall alverine-related exposure (based on AUC_0-t_ data) are presented in Figure 2.

Very high coefficients of variation were noted for AUC_0-t_ data of alverine parent (209%) as well as metabolites 4-hydroxy alverine (111%) and N-desethyl alverine (163%). A statistical test for identification of outliers was subsequently applied to the exploratory study data in order to identify the subjects contributing to the high variability observed and to check with which metabolic step the outlying behavior interfered. The test identified two (out of twelve) subjects with extreme outlying behavior (observations outside 3^rd^ Quantile +3 x Interquartile Range) specific to the hydroxylation pathway (both subjects being identified as outliers in the parent alverine and 4-hydroxy alverine analysis, while no statistically significant differences from the mean of the general population were noted for 4-hydroxy alverine glucuronide or N-desethyl-alverine). The total exposure of circulating unchanged alverine was 122 and respectively 199 times higher for the two outlying subjects as compared to the regular metabolizers within the study population. For the two poor hydroxylators, abundant availability of unchanged product for minor metabolic pathways has also translated into higher than average concentrations of N-desethyl alverine (though not statistically significant based on the applied outliers test). The same pattern of behavior was noted also in a different metabolic profiling study conducted in house (data not authorized for publication yet) where the incidence of poor alverine hydroxylators in Caucasian subjects was 21% (three out of fourteen subjects).

The cause for the different metabolic behavior noted in outliers could not be linked to any external factors. All clinical laboratory parameters determined for the outlying subjects revealed unremarkable results and none of them experienced adverse events nor took any medication during the two weeks prior to dosing or throughout the clinical part of the study. All subjects were non-smokers so tobacco-mediated enzymatic inhibition/induction can also be excluded. No CYP-inhibiting/inducing foods or drinks were consumed during the 72 h before administration or throughout the clinical part of the study.

Being a weak base and highly lipophilic (logP = 5.46 (Alverine citrate Drug Bank Monograph, 2020)), although administered as citrate salt, one of the possible explanations for the observed pharmacokinetic behavior could be the tendency of aliverine to precipitate at intestinal pH. Biopharmacists consider this a frequently met phenomenon (Mircioiu et al., 2012; Preda et al., 2012) typically resulting in a high variability in absorption. All lipophilic drugs are extensively metabolized, the pharmacokinetics of both parent compound and metabolites being highly variable, and as a result, some subjects behave as outliers due to a combination of variability in intestinal solubility (Sandulovici et al., 2009; Mircioiu et al., 2010; Tandel et al., 2013; Sandulovici et al., 2020) and genetic polymorphism. The type of genetic polymorphism affecting the activities of drug-metabolizing enzymes in the case of alverine citrate remains yet unidentified (more specific information not available as the subjects were not screened for CYP- phenotype or -genotype), however the effect observed in outliers was a decrease in the magnitude of an otherwise significant first-pass effect.

Despite the high variability in hydroxylation, the subsequent glucuronidation step was observed to be a stable and abundant metabolic process. The coefficient of variation for 4-hydroxy alverine glucuronide over the entire study population was only 45%, there were no subjects with outlying AUC_0-t_ data, and the metabolite represented about 94% of alverine-derived moieties circulating (see Figure 2).

The metabolism of alverine, apart from being extensive, was also noted to be fast (0.75 h median T_max_ for unchanged alverine and 1.00 h median T_max_ for the metabolites 4-hydroxy alverine, 4- hydroxy alverine glucuronide and N-desethyl alverine).

### Safety Results

One adverse event occurred during the present study (mild dizziness, lasting 12 min). The medication Spasmonal^®^ Forte 120 mg hard capsule, administered as single dose in fasting state, was very well tolerated by the healthy volunteers participating in this study.

## Discussion

The first HPLC-MS/MS analytical protocol for determination of alverine parent, 4-hydroxy alverine, N-desethyl alverine (detected simultaneously with one analytical method) and 4-hydroxy alverine glucuronide (separately, with a different analytical method) in human plasma was developed and validated according to current regulatory requirements.

These validated methods were then applied for analyzing plasma samples collected during an open label, non-comparative, single dose, one-period, one-treatment, pharmacokinetic and metabolic profile study of Spasmonal^®^ Forte 120 mg hard capsule, conducted in 12 fasting healthy male and female volunteers.

The study confirmed previous observations that pharmacokinetic variability of parent alverine far exceeds the expectations one would get upon review of literature data, albeit from studies conducted on Indian population (Gomes et al., 2009; Ghosh et al., 2010; Seelam et al., 2015; Rathod et al., 2017). The metabolic process most susceptible to outlying performance in Caucasian population was found to be hydroxylation to the active metabolite 4-hydroxy alverine. Exogenous factors excluded, it is believed that the poor hydroxylator status observed in about 17% of the study population could stem from either biopharmaceutical properties of the formulation, or, most likely, genetic polymorphism (as this would explain also the inconsistencies in degree of variability as compared to the studies conducted on Indian population).

Another interesting aspect revealed was that alverine parent accounts for only 3%, whereas total 4-hydroxy alverine (free and conjugated) accounts for about 94% of alverine-related moieties in circulation (based on comparisons of total exposure). This finding would strongly suggest that the research and development program for future generic alverine formulations would benefit from integrating pharmacokinetic metabolite data in both IVIVC (Savu et al., 2016; Mircioiu et al., 2019) and *in vivo* bioequivalence testing models.

Safety data collected during the study permitted to conclude that alverine citrate, administered in single 120 mg dose to healthy volunteers, was very well tolerated. This observation is line with the known good tolerability profile of the molecule, as ascertained in larger clinical studies (Wittmann et al., 2010; Martínez-Vázquez et al., 2012; Ducrotte et al., 2014).

Pharmacokinetic data collected during the study permitted to conclude that alverine undergoes extensive and fast metabolism.

## Data Availability Statement

The raw data supporting the conclusions of this article will be made available by the authors, without undue reservation.

## Ethics Statement

The studies involving human participants were reviewed and approved by the Moldavian National Ethics Committee. The “healthy volunteers” provided their written informed consent to participate in this study.

## Author Contributions

SR-S and SD were involved in the clinical and analytical conduct of the study. All three authors contributed to the data analysis process and drafting of the manuscript.

## Conflict of Interest

SR-S is employed by the company 3S-Pharmacological Consultation & Research GmbH and author SD is employed by the company 3SPharmacological Consultation & Res. SRL.

The remaining author declares that the research was conducted in the absence of any commercial or financial relationships that could be construed as a potential conflict of interest.

The reviewer CG declared a past collaboration with one of the authors RS to the handling editor.
