# Supplementary figures and images for: Single Dose Study Assessing the Pharmacokinetic and Metabolic Profile of Alverine Citrate in Healthy Volunteers
Source: Front Pharmacol. 2021 Jan 20;11:620451. doi: 10.3389/fphar.2020.620451 (PMC7855969; doi:10.3389/fphar.2020.620451)

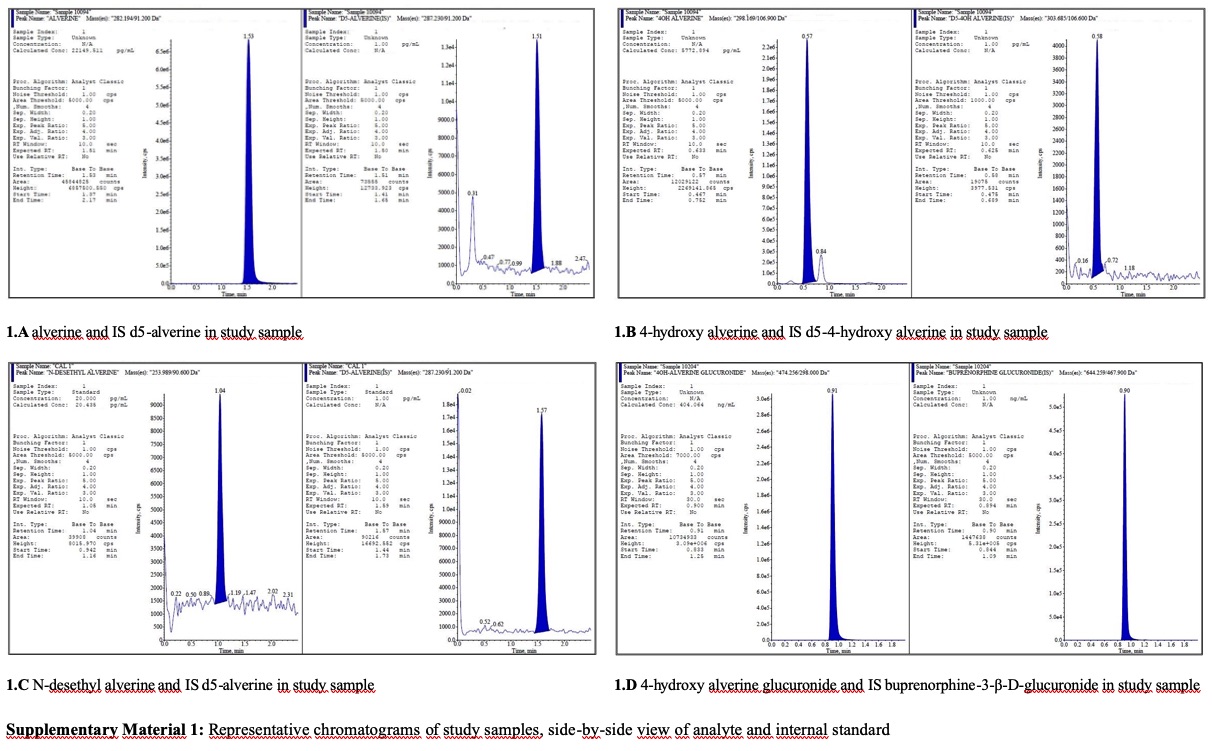

Supplement: Supplementary file 1 [file image1.jpeg]
